# Tetra­aqua­tetra­manganese(II) *catena*-[germanodihydroxidodi(hydrogen­phosphate)diphosphate]

**DOI:** 10.1107/S160053681201673X

**Published:** 2012-04-25

**Authors:** Xin Zhang, Lei Wen, Hong-Ming Chen, Jin-Xiao Mi, Ya-Xi Huang

**Affiliations:** a Department of Materials Science and Engineering, College of Materials, Xiamen University, Xiamen 361005, Fujian Province, People’s Republic of China

## Abstract

The title compound, Mn_4_(H_2_O)_4_[Ge(OH)_2_(HPO_4_)_2_(PO_4_)_2_], was synthesized by the solvothermal method. Its crystal structure is isotypic with the iron and cobalt analogues [Huang *et al.* (2012 ▶). *Inorg. Chem.* **51**, 3316–3323]. In the crystal structure, the framework is built from undulating manganese phosphate sheets parallel to (010) inter­connected by GeO_6_ octa­hedra (at the inversion center), resulting in a three-dimensional network with eight-membered ring channels into which all the protons point. The undulating manganese phosphate sheet consists of zigzag manganese octa­hedral chains along [10-1], built from MnO_4_(OH)(OH_2_) octa­hedra and MnO_5_(OH_2_) octa­hedra by sharing their *trans* or skew edges, which are inter­connected by PO_3_(OH) and PO_4_ tetra­hedra *via* corner-sharing. The crystal structure features extensive O—H⋯O hydrogen-bonding inter­actions.

## Related literature

For background to germanophosphates, see: Brock *et al.* (1998 ▶); Corma (1997 ▶); Li *et al.* (2000 ▶); Liu, Yang, Wang *et al.* (2008 ▶); Liu, Yang, Zhang *et al.* (2008 ▶); Zhao *et al.* (2009 ▶); Zubieta (1994 ▶). For isotypic structures of the Fe^II^ and Co^II^ analogues, see: Huang *et al.* (2012 ▶).

## Experimental

### 

#### Crystal data

Mn_4_(H_2_O)_4_[Ge(OH)_2_(HPO_4_)_2_(PO_4_)_2_]
*M* *_r_* = 780.33
Monoclinic, 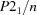

*a* = 6.5336 (3) Å
*b* = 16.3869 (7) Å
*c* = 7.7048 (3) Å
β = 98.506 (4)°
*V* = 815.84 (6) Å^3^
*Z* = 2
Mo *K*α radiation
μ = 5.35 mm^−1^
*T* = 173 K
0.10 × 0.08 × 0.05 mm

#### Data collection

Oxford Diffraction CCD area-detector diffractometer
Absorption correction: multi-scan (*CrysAlis RED*; Oxford Diffraction, 2005 ▶) *T*_min_ = 0.604, *T*_max_ = 0.765
5173 measured reflections
2215 independent reflections
1808 reflections with *I* > 2σ(*I*)
*R*_int_ = 0.049

#### Refinement

*R*[*F* ^2^ > 2σ(*F* ^2^)] = 0.042
*wR*(*F* ^2^) = 0.064
*S* = 1.11
2215 reflections
158 parameters
5 restraints
H atoms treated by a mixture of independent and constrained refinement
Δρ_max_ = 0.67 e Å^−3^
Δρ_min_ = −0.71 e Å^−3^

### 

Data collection: *CrysAlis CCD* (Oxford Diffraction, 2005 ▶); cell refinement: *CrysAlis CCD*; data reduction: *CrysAlis RED* (Oxford Diffraction, 2005 ▶); program(s) used to solve structure: *SHELXS97* (Sheldrick, 2008 ▶); program(s) used to refine structure: *SHELXL97* (Sheldrick, 2008 ▶); molecular graphics: *DIAMOND* (Brandenburg, 2005 ▶) and *ATOMS* (Dowty, 2004 ▶); software used to prepare material for publication: *SHELXL97* and *WinGX* (Farrugia, 1999 ▶).

## Supplementary Material

Crystal structure: contains datablock(s) I, global. DOI: 10.1107/S160053681201673X/br2199sup1.cif

Structure factors: contains datablock(s) I. DOI: 10.1107/S160053681201673X/br2199Isup2.hkl

Additional supplementary materials:  crystallographic information; 3D view; checkCIF report

## Figures and Tables

**Table 1:** Hydrogen-bond geometry (Å, °)

*D*—H⋯*A*	*D*—H	H⋯*A*	*D*⋯*A*	*D*—H⋯*A*
O1—H1⋯O9^i^	0.82 (1)	2.49 (5)	3.039 (5)	126 (5)
O1—H1⋯O7^i^	0.82 (1)	2.60 (5)	3.166 (4)	127 (5)
O1—H2⋯O8^ii^	0.82 (1)	2.33 (3)	3.101 (4)	157 (6)
O1—H2⋯O11^iii^	0.82 (1)	2.39 (5)	2.878 (4)	119 (5)
O2—H3⋯O9^iv^	0.82 (1)	2.01 (3)	2.733 (4)	147 (5)
O2—H4⋯O1^iii^	0.82 (1)	2.03 (3)	2.765 (5)	149 (6)
O2—H3⋯O10^v^	0.82 (1)	2.63 (5)	3.161 (4)	123 (5)
O8—H5⋯O10^vi^	0.83	2.11	2.692 (4)	126 (1)
O9—H6⋯O5^vii^	0.82 (1)	1.80 (2)	2.584 (4)	160 (6)

Symmetry codes: (i) 
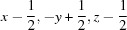
; (ii) 
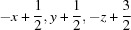
; (iii) 
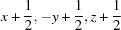
; (iv) 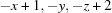; (v) 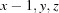; (vi) 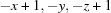; (vii) 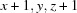.

**Fig. 1.**
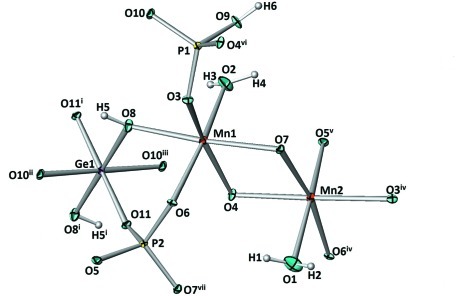
Asymmetric unit of the title compound, showing the coordination environments of Ge, Mn and P atoms. Thermal ellipsoids are drawn at the 50% probability level. Symmetry codes: (i) –x, –y, –z+1; (ii) –x+1, –y, –z+1; (iii) x–1, y, z; (iv) x–1/2, –y+1/2, z+1/2; (v) x, y, z+1; (vi) x+1, y, z; (vii) x–1/2, –y+1/2, z–1/2.

**Fig. 2.**
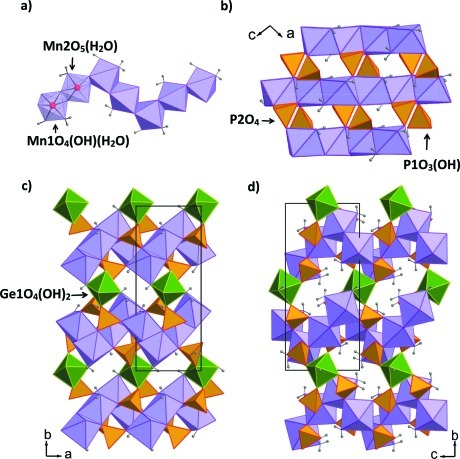
Polyhedral presentation of the title compound, a) manganese octahedra share their trans or skew edges to form zigzag chains parallel to [10-1], b) these manganese octahedral chains are linked by phosphate tetrahedra via common O-corners to form undulating manganese phosphate sheets parallel to (010), c) the undulating sheets are further connected by GeO4(OH)2 octahedra along [010], resulting in a three-dimensional network structure, d) the network structure comprises 8-membered ring channels running along [100] direction where all the protons protrude to. MnO6 octahedra: purple, GeO6 octahedra: green, PO4 tetrahedra: orange, H atoms: light grey spheres.

## supplementary crystallographic information

### Comment

Open-framework compounds attract much attention due to their rich structural chemistry and potential catalytic, electrical, optical, and magnetic properties (Brock *et al.*, 1998; Corma, 1997; Zubieta, 1994). Germanophosphates, as one kind of open-framework compounds, have not been fully explored, and only a few compounds had been synthesized up to date (Li *et al.*, 2000; Liu, Yang, Wang *et al.*, 2008; Liu, Yang, Zhang *et al.*, 2008; Zhao *et al.*, 2009). During our systematical investigation on the transition metal germanophosphate system, we obtain the first manganese compound, Mn^II^_4_(H_2_O)_4_[Ge(OH)_2_(HPO_4_)_2_(PO_4_)_2_], which is isostructural with its Fe and Co-analogues (Huang *et al.*, 2012) published recently.

The asymmetric unit of the title compound consists of two distinct Mn atoms, one Ge atom, and two crystallographically independent P atoms (Figure 1). Mn1 is surrounding by four O atoms, an OH-group, and a H_2_O molecule forming a distorted octahedron Mn1O_4_(OH)(OH_2_) with bond distances of 2.159-2.207 Å. Mn2 adopt a nearly regular octahedral coordination to five O atoms and a water molecule with bond distances ranging from 2.176 to 2.224 Å. Ge atom coordinates to four O-atoms in the equatorial plane with shorter bond lengths of 1.846-1.871 Å and two hydroxyl groups in trans-position with longer distance of 1.913 Å. Both P atoms are in tetrahedral coordination with bond lengths of 1.526-1.573 Å. All the bond lengths and angles of the title compound are similar to those of the known germanophosphates (Huang *et al.*, 2012; Liu, Yang, Wang *et al.*, 2008; Liu, Yang, Zhang *et al.*, 2008; Zhao *et al.*, 2009). The Mn2O_5_(OH_2_) octahedra share their trans-edges with two adjacent Mn1O_4_(OH)(OH_2_) octahedra, in turn, Mn1-octahedra share skew-edges to two neighboring Mn2-octahedra, resulting in a zigzag chain running along [10-1] (Figure 2a). These manganese octahedral chains are further linked by the P1O_3_(OH) and P2O_4_ tetrahedra through corner sharing to form undulating sheets (Figure 2b). These sheets are further interconnected by GeO_4_(OH)_2_ octahedra, sitting at the inverse center, along [010] via common O-corners (Figure 2c) to form a neutral 3-D network with 8-membered ring channels parallel to [100] where all the protons protrude to (Figure 2d). Compared with the two isotypic Fe- and Co-compounds, the Mn-O distances are longer than those of Co- and Fe-compound which is consistent to the longer ionic radius of Mn than those of Fe and Co analogues.

### Experimental

The crystals of the title compound were solvothermally synthesized by a mixture of GeO_2_ (0.073 g), Mn(CH_3_COO)_2_.4H_2_O (0.171 g), H_3_PO_4_(85 wt%, 1 mL), H_2_O (2 mL), triethylamine (1 mL), and 1,2-propanediol (2 mL). The mixture was transferred into a Teflon-lined stainless steel autoclave (internal volume of 15 mL) and heated at 423K for 7 days under static conditions. After the autoclaves cooling to room temperature, the products were filtered off and washed by distilled water for several times and dried in air. Finally light-pink block-shaped crystals were obtained. It is needed to note that the products often contain a minor impurity of GeO_2_. Many efforts have been made to get pure phase. However, even the pure phase was obtained but the yield is so low that not enough for further characterization. Thus only single crystal X-ray diffraction of the title compound was carried out.

### Refinement

Originally, all hydrogen positions were located from the difference Fourier map and refined by applying the constraint of displacement parameters as 0.05 eÅ^3^ and a bond distance of *d*(O–H) = 0.82 (1)Å. After the refinement, O8-H5 turned out to have no acceptor atom. Therefore, the H5 position was calculated geometrically and fixed without applying further refinement.

### Crystal data

**Table:**

Mn_4_(H_2_O)_4_[Ge(OH)_2_(HPO_4_)_2_(PO_4_)_2_]	*Z* = 2
*M**_r_* = 780.33	*F*(000) = 760
Monoclinic, *P*2_1_/*n*	*D*_x_ = 3.176 Mg m^−^^3^
Hall symbol: -P 2yn	Mo *K*α radiation, λ = 0.71073 Å
*a* = 6.5336 (3) Å	θ = 3.0–32.5°
*b* = 16.3869 (7) Å	µ = 5.35 mm^−^^1^
*c* = 7.7048 (3) Å	*T* = 173 K
β = 98.506 (4)°	Block-shaped, light pink
*V* = 815.84 (6) Å^3^	0.10 × 0.08 × 0.05 mm

### Data collection

**Table:**

Oxford Diffraction CCD area-detector diffractometer	2215 independent reflections
Radiation source: fine-focus sealed tube	1808 reflections with *I* > 2σ(*I*)
Graphite monochromator	*R*_int_ = 0.049
326 images,Δω=1°, Exp time: 40 s. scans	θ_max_ = 30.7°, θ_min_ = 3.0°
Absorption correction: multi-scan (*CrysAlis CCD*; Oxford Diffraction, 2005)	*h* = −8→8
*T*_min_ = 0.604, *T*_max_ = 0.765	*k* = −23→21
5173 measured reflections	*l* = −10→5

### Refinement

**Table:**

Refinement on *F*^2^	Secondary atom site location: difference Fourier map
Least-squares matrix: full	Hydrogen site location: inferred from neighbouring sites
*R*[*F*^2^ > 2σ(*F*^2^)] = 0.042	H atoms treated by a mixture of independent and constrained refinement
*wR*(*F*^2^) = 0.064	*w* = 1/[σ^2^(*F*_o_^2^) + (0.007*P*)^2^] where *P* = (*F*_o_^2^ + 2*F*_c_^2^)/3
*S* = 1.11	(Δ/σ)_max_ < 0.001
2215 reflections	Δρ_max_ = 0.67 e Å^−^^3^
158 parameters	Δρ_min_ = −0.71 e Å^−^^3^
5 restraints	Extinction correction: *SHELXL97* (Sheldrick, 2008), Fc^*^=kFc[1+0.001xFc^2^λ^3^/sin(2θ)]^-1/4^
Primary atom site location: structure-invariant direct methods	Extinction coefficient: 0.0025 (3)

### Special details

**Table:**

Geometry. All esds (except the esd in the dihedral angle between two l.s. planes) are estimated using the full covariance matrix. The cell esds are taken into account individually in the estimation of esds in distances, angles and torsion angles; correlations between esds in cell parameters are only used when they are defined by crystal symmetry. An approximate (isotropic) treatment of cell esds is used for estimating esds involving l.s. planes.
Refinement. Refinement of F^2^ against ALL reflections. The weighted R-factor wR and goodness of fit S are based on F^2^, conventional R-factors R are based on F, with F set to zero for negative F^2^. The threshold expression of F^2^ > 2sigma(F^2^) is used only for calculating R-factors(gt) etc. and is not relevant to the choice of reflections for refinement. R-factors based on F^2^ are statistically about twice as large as those based on F, and R- factors based on ALL data will be even larger.

### Fractional atomic coordinates and isotropic or equivalent isotropic displacement parameters (Å^2^)

**Table:**

	*x*	*y*	*z*	*U*_iso_*/*U*_eq_	
Ge1	0.0000	0.0000	0.5000	0.00570 (15)	
Mn1	0.33624 (9)	0.13618 (4)	0.73967 (8)	0.00616 (15)	
Mn2	0.10405 (9)	0.27193 (4)	0.97488 (8)	0.00687 (15)	
P1	0.85555 (15)	0.10913 (6)	0.78765 (14)	0.0048 (2)	
P2	0.06543 (15)	0.18774 (6)	0.37557 (14)	0.0048 (2)	
O1	0.0486 (5)	0.3570 (2)	0.7515 (5)	0.0212 (8)	
O2	0.3396 (5)	0.05675 (19)	0.9714 (4)	0.0169 (7)	
O3	0.6508 (4)	0.13783 (16)	0.6846 (4)	0.0068 (6)	
O4	0.0282 (4)	0.17242 (16)	0.7897 (4)	0.0075 (6)	
O5	0.0928 (4)	0.18148 (16)	0.1813 (4)	0.0071 (6)	
O6	0.2743 (4)	0.20020 (16)	0.4915 (4)	0.0063 (6)	
O7	0.4116 (4)	0.24382 (15)	0.9032 (4)	0.0071 (6)	
O8	0.2712 (4)	0.02156 (17)	0.5966 (4)	0.0099 (6)	
O9	0.8236 (4)	0.09402 (17)	0.9835 (4)	0.0094 (6)	
O10	0.9176 (4)	0.02614 (16)	0.7215 (4)	0.0078 (6)	
O11	−0.0403 (4)	0.10841 (16)	0.4270 (4)	0.0074 (6)	
H1	0.101 (8)	0.336 (3)	0.672 (5)	0.050*	
H2	0.117 (8)	0.399 (2)	0.768 (8)	0.050*	
H3	0.252 (6)	0.020 (2)	0.959 (8)	0.050*	
H4	0.364 (9)	0.076 (3)	1.070 (3)	0.050*	
H5	0.296	0.014	0.495	0.050*	
H6	0.917 (6)	0.111 (3)	1.056 (6)	0.050*	

### Atomic displacement parameters (Å^2^)

**Table:**

	*U*^11^	*U*^22^	*U*^33^	*U*^12^	*U*^13^	*U*^23^
Ge1	0.0067 (3)	0.0038 (3)	0.0065 (3)	−0.0004 (2)	0.0006 (2)	−0.0006 (2)
Mn1	0.0059 (3)	0.0056 (3)	0.0069 (3)	0.0000 (2)	0.0010 (2)	−0.0001 (2)
Mn2	0.0075 (3)	0.0062 (3)	0.0069 (3)	−0.0002 (2)	0.0011 (2)	−0.0009 (3)
P1	0.0050 (5)	0.0048 (5)	0.0048 (5)	−0.0003 (4)	0.0008 (4)	0.0003 (4)
P2	0.0048 (5)	0.0038 (5)	0.0056 (5)	0.0009 (4)	0.0006 (4)	0.0006 (4)
O1	0.0201 (19)	0.024 (2)	0.0185 (19)	−0.0062 (15)	−0.0014 (15)	0.0063 (16)
O2	0.0222 (18)	0.0140 (18)	0.0122 (17)	−0.0081 (13)	−0.0054 (15)	0.0033 (14)
O3	0.0040 (13)	0.0076 (15)	0.0088 (15)	−0.0006 (11)	0.0005 (12)	0.0015 (12)
O4	0.0070 (14)	0.0081 (15)	0.0083 (15)	−0.0002 (11)	0.0043 (12)	−0.0025 (11)
O5	0.0092 (14)	0.0059 (15)	0.0061 (15)	−0.0004 (11)	0.0003 (12)	0.0024 (11)
O6	0.0042 (13)	0.0082 (15)	0.0061 (15)	0.0010 (11)	−0.0010 (11)	0.0011 (11)
O7	0.0080 (14)	0.0040 (14)	0.0090 (15)	0.0000 (11)	0.0000 (12)	−0.0013 (12)
O8	0.0097 (14)	0.0117 (16)	0.0087 (15)	−0.0027 (11)	0.0023 (12)	−0.0027 (12)
O9	0.0110 (15)	0.0136 (16)	0.0031 (15)	−0.0034 (12)	−0.0009 (12)	0.0003 (12)
O10	0.0119 (14)	0.0037 (14)	0.0078 (15)	0.0001 (11)	0.0018 (12)	−0.0005 (11)
O11	0.0089 (14)	0.0038 (14)	0.0092 (15)	0.0008 (11)	0.0000 (12)	0.0004 (12)

### Geometric parameters (Å, º)

**Table:**

Ge1—O8^i^	1.851 (3)	Mn2—O7	2.210 (3)
Ge1—O8	1.851 (3)	Mn2—O6^iv^	2.225 (3)
Ge1—O11^i^	1.870 (3)	P1—O3	1.525 (3)
Ge1—O11	1.870 (3)	P1—O10	1.528 (3)
Ge1—O10^ii^	1.913 (3)	P1—O4^vi^	1.531 (3)
Ge1—O10^iii^	1.913 (3)	P1—O9	1.573 (3)
Ge1—H5	1.9535	P2—O6	1.530 (3)
Mn1—O3	2.159 (3)	P2—O5	1.537 (3)
Mn1—O6	2.166 (3)	P2—O7^vii^	1.542 (3)
Mn1—O7	2.181 (3)	P2—O11	1.551 (3)
Mn1—O4	2.186 (3)	O1—H1	0.817 (10)
Mn1—O8	2.188 (3)	O1—H2	0.818 (10)
Mn1—O2	2.207 (3)	O2—H3	0.822 (10)
Mn2—O4	2.175 (3)	O2—H4	0.815 (10)
Mn2—O3^iv^	2.178 (3)	O8—H5	0.83
Mn2—O5^v^	2.183 (3)	O9—H6	0.816 (10)
Mn2—O1	2.203 (4)		
			
O8^i^—Ge1—O8	180.00 (7)	O5^v^—Mn2—O7	99.81 (10)
O8^i^—Ge1—O11^i^	91.35 (12)	O1—Mn2—O7	89.26 (12)
O8—Ge1—O11^i^	88.65 (12)	O4—Mn2—O6^iv^	93.62 (10)
O8^i^—Ge1—O11	88.65 (12)	O3^iv^—Mn2—O6^iv^	81.31 (10)
O8—Ge1—O11	91.35 (12)	O5^v^—Mn2—O6^iv^	87.72 (10)
O11^i^—Ge1—O11	180.0	O1—Mn2—O6^iv^	82.32 (12)
O8^i^—Ge1—O10^ii^	91.28 (12)	O7—Mn2—O6^iv^	168.93 (11)
O8—Ge1—O10^ii^	88.72 (12)	O3—P1—O10	110.87 (15)
O11^i^—Ge1—O10^ii^	89.43 (12)	O3—P1—O4^vi^	112.63 (16)
O11—Ge1—O10^ii^	90.57 (12)	O10—P1—O4^vi^	112.02 (16)
O8^i^—Ge1—O10^iii^	88.72 (12)	O3—P1—O9	108.50 (16)
O8—Ge1—O10^iii^	91.28 (12)	O10—P1—O9	105.01 (16)
O11^i^—Ge1—O10^iii^	90.57 (12)	O4^vi^—P1—O9	107.40 (16)
O11—Ge1—O10^iii^	89.43 (12)	O6—P2—O5	110.80 (16)
O10^ii^—Ge1—O10^iii^	180.0	O6—P2—O7^vii^	111.45 (15)
O3—Mn1—O6	83.08 (10)	O5—P2—O7^vii^	110.81 (16)
O3—Mn1—O7	87.89 (10)	O6—P2—O11	110.82 (15)
O6—Mn1—O7	96.60 (10)	O5—P2—O11	108.25 (16)
O3—Mn1—O4	163.50 (10)	O7^vii^—P2—O11	104.49 (16)
O6—Mn1—O4	88.13 (10)	H1—O1—H2	101 (6)
O7—Mn1—O4	79.25 (10)	H3—O2—H4	115 (6)
O3—Mn1—O8	91.79 (10)	P1—O3—Mn1	132.90 (17)
O6—Mn1—O8	88.50 (10)	P1—O3—Mn2^viii^	127.70 (16)
O7—Mn1—O8	174.81 (11)	Mn1—O3—Mn2^viii^	96.96 (10)
O4—Mn1—O8	101.94 (10)	P1^ii^—O4—Mn2	127.63 (17)
O3—Mn1—O2	105.70 (12)	P1^ii^—O4—Mn1	120.69 (15)
O6—Mn1—O2	167.96 (11)	Mn2—O4—Mn1	101.13 (10)
O7—Mn1—O2	92.02 (11)	P2—O5—Mn2^ix^	133.27 (16)
O4—Mn1—O2	85.22 (12)	P2—O6—Mn1	119.00 (15)
O8—Mn1—O2	83.08 (11)	P2—O6—Mn2^viii^	141.41 (17)
O4—Mn2—O3^iv^	171.44 (11)	Mn1—O6—Mn2^viii^	95.39 (10)
O4—Mn2—O5^v^	86.56 (10)	P2^x^—O7—Mn1	127.21 (17)
O3^iv^—Mn2—O5^v^	86.36 (10)	P2^x^—O7—Mn2	121.22 (15)
O4—Mn2—O1	88.11 (12)	Mn1—O7—Mn2	100.20 (10)
O3^iv^—Mn2—O1	97.96 (12)	Ge1—O8—Mn1	118.00 (13)
O5^v^—Mn2—O1	168.39 (12)	P1—O10—Ge1^vi^	128.35 (17)
O4—Mn2—O7	78.87 (10)	P2—O11—Ge1	145.55 (17)
O3^iv^—Mn2—O7	107.09 (10)		

Symmetry codes: (i) −*x*, −*y*, −*z*+1; (ii) *x*−1, *y*, *z*; (iii) −*x*+1, −*y*, −*z*+1; (iv) *x*−1/2, −*y*+1/2, *z*+1/2; (v) *x*, *y*, *z*+1; (vi) *x*+1, *y*, *z*; (vii) *x*−1/2, −*y*+1/2, *z*−1/2; (viii) *x*+1/2, −*y*+1/2, *z*−1/2; (ix) *x*, *y*, *z*−1; (x) *x*+1/2, −*y*+1/2, *z*+1/2.

### Hydrogen-bond geometry (Å, º)

**Table:**

*D*—H···*A*	*D*—H	H···*A*	*D*···*A*	*D*—H···*A*
O1—H1···O9^vii^	0.82 (1)	2.49 (5)	3.039 (5)	126 (5)
O1—H1···O7^vii^	0.82 (1)	2.60 (5)	3.166 (4)	127 (5)
O1—H2···O8^xi^	0.82 (1)	2.33 (3)	3.101 (4)	157 (6)
O1—H2···O11^x^	0.82 (1)	2.39 (5)	2.878 (4)	119 (5)
O2—H3···O9^xii^	0.82 (1)	2.01 (3)	2.733 (4)	147 (5)
O2—H4···O1^x^	0.82 (1)	2.03 (3)	2.765 (5)	149 (6)
O2—H3···O10^ii^	0.82 (1)	2.63 (5)	3.161 (4)	123 (5)
O8—H5···O10^iii^	0.83	2.11	2.692 (4)	126 (1)
O9—H6···O5^xiii^	0.82 (1)	1.80 (2)	2.584 (4)	160 (6)

Symmetry codes: (ii) *x*−1, *y*, *z*; (iii) −*x*+1, −*y*, −*z*+1; (vii) *x*−1/2, −*y*+1/2, *z*−1/2; (x) *x*+1/2, −*y*+1/2, *z*+1/2; (xi) −*x*+1/2, *y*+1/2, −*z*+3/2; (xii) −*x*+1, −*y*, −*z*+2; (xiii) *x*+1, *y*, *z*+1.
